# Molecular diagnosis and characterization of *Anaplasma marginale* and *Ehrlichia ruminantium* infecting beef cattle of Maputo Province, Mozambique

**DOI:** 10.1186/s12917-024-04045-4

**Published:** 2024-05-10

**Authors:** Carlos António Matos, Cesária Fiossiane Nomboro, Luiz Ricardo Gonçalves, Aida Cristina Cala, Carlos Francisco Sitoe, Ana Paula Rúpia Vinte, Cristovão Mario Mondlane, Marcos Rogério André, Maria do Carmo Carrilho

**Affiliations:** 1 Laboratório de Parasitologia, Direcção de Ciências Animais, Avenida de Moçambique, km 1.5, Bairro do Jardim, Cidade de Maputo, C.P. 1922 Moçambique; 2 Universidade Pedagógica Maputo, Maputo, Moçambique; 3 Imunodot Diagnósticos Veterinários – IMUNODOT, Jaboticabal, SP Brasil; 4 Vector-Borne Bioagents Laboratory (VBBL), Departamento de Patologia, Reprodução e Saúde Única, São Paulo State University (UNESP), School of Agricultural and Veterinary Sciences, Jaboticabal, Jaboticabal, SP Brasil

**Keywords:** *Anaplasma* spp., *Ehrlichia ruminantium*, Cattle, 16S rRNA, *Map*1, *msp5*, Maputo Province

## Abstract

**Background:**

Members of the Anaplasmataceae family, such as the *Anaplasma* and *Ehrlichia* species, cause economic losses and public health risks. However, the exact economic impact has not been comprehensively assessed in Mozambique due to limited data available on its basic epidemiology. Therefore, we investigated the molecular occurrence and identity of *Anaplasma* and *Ehrlichia* spp. infecting beef cattle in Maputo province, Mozambique.

**Methods:**

A total of 200 whole blood samples were collected from apparently healthy beef cattle. Whole blood DNA was extracted and tested for presence of *Anaplasma* spp. and *Ehrlichia ruminantium* DNA through amplification of the 16S rRNA and *map1* genes. Positive samples to *Anaplasma* spp. were subject to PCR assay targeting the *A. marginale*-*msp5* gene. Amplicons obtained were purified, sequenced and subject to phylogenetic analyses.

**Results:**

*Anaplasma* spp., *A. marginale* and *E*. *ruminantium* were detected in 153 (76.5%), 142 (71%) and 19 (9.5%) of all the samples analyzed, respectively*.* On this same sample group, 19 (9.5%) were co-infected with *A. marginale* and *E*. *ruminantium*. The 16S rRNA sequences of *Anaplasma* spp. obtained were phylogenetically related to *A*. *marginale*, *A*. *centrale* and *A. platys*. Phylogenetic analysis revealed that *A. marginale*-*msp5* nucleotide sequences were grouped with sequences from Asia, Africa and Latin America, whereas *E*. *ruminantium*-*map1* DNA nucleotide sequences were positioned in multiple clusters.

**Conclusion:**

Cattle in Maputo Province are reservoirs for multiple *Anaplasma* species. A high positivity rate of infection by *A. marginale* was observed, as well as high genetic diversity of *E. ruminantium*. Furthermore, five new genotypes of *E. ruminantium*-*map1* were identified.

## Introduction

Tick and tick-borne diseases are one of the most significant constraints on livestock production in Mozambique. Diseases such as East Coast fever (caused by *Theileria parva*), heartwater (caused by *Ehrlichia ruminantium*), anaplasmosis (caused by *Anaplasma marginale*) and babesiosis (caused by *Babesia bigemina* and *Babesia bovis*) cause significant economic losses for cattle in the country [1–4]. Infections by Anaplasmataceae agents produce significant economic losses in Africa, where roughly 150 million animals are susceptible to infection [5, 6]. Mozambique holds the highest pooled prevalence estimate of tick-borne pathogens in domestic animals across the Southern African Developing Community (SADC), which has warm subtropical and tropical climates [7].

*Anaplasma* spp. and *Ehrlichia* spp. are tick-borne obligate intracellular Gram-negative bacteria that infects hematopoietic cells and belongs to Anaplasmataceae family. These species are of veterinary and public health significance [8–10] and are maintained in nature through enzootic cycles that includes Ixodidae ticks and vertebrate hosts [11]. Animals that recover from infection act as long-lasting carriers, with a small number of infected erythrocytes. These carrier animals play a significant role in the transmission of these tick-borne infections [12].

The Anaplasmataceae family comprises four main genera, namely *Anaplasma, Ehrlichia*, *Neorickettsia* and *Wolbachia* [12]. The genus *Anaplasma* comprises seven species, namely *Anaplasma marginale*, *A. bovis*, *A. centrale*, *A. ovis*, *A. phagocytophilum, A. capra* and *A. platys*. The genus *Ehrlichia* consists of six species, namely *Ehrlichia canis*, *E. chaffeensis*, *E. ewingii*, *E. muris, E. minasensis* and *E. ruminantium* [13, 14].

*Anaplasma* species known to infect domestic ruminants including cattle are *A. marginale*, *A. bovi*s, *A. capra*, *A. centrale*, *A. ovis*, *A. phagocytophilum,* and *A. platys* [15–17]. Additionally, three other putative novel species of *Anaplasma* were recently detected in cattle in Ethiopia, namely *Anaplasma* sp*.* Hadesa, *Anaplasma* sp. Dedessa, and *Anaplasma* sp. Saso [18]. When it comes to *Ehrlichia* genus, *E. ruminantium* is the most common species known to infect cattle [19]. A new *Ehrlichia,* namely *Ehrlichia minasensis,* was detected in *Rhipicephalus microplus* ticks and cattle in Brazil, Ethiopia and Kenya [18, 20, 21], causing clinical signs similar to those of canine ehrlichiosis in an experimentally infected calf [22].

Anaplasmosis is caused by several *Anaplasma* species and is responsible for significant challenges for animal breeders. Indeed, infection by *Anaplasma* spp. increases the costs for veterinary care since it causes a reduction in animal body weight, decrease in milk production, abortions, and often death [23, 24]. *Anaplasma marginale* is the main causative agent of bovine anaplasmosis worldwide. This species is biologically transmitted by approximately 20 tick species and mechanically transmitted by biting flies and blood-contaminated fomites [24, 25]. The disease is characterized by anemia, weight loss, abortion, and death, resulting in significant economic losses for the cattle industry [23, 25, 26].

*Ehrlichia ruminantium* is the etiological agent of heartwater disease in domestic ruminants and is transmitted by *Amblyomma* ticks [27]. The disease is limited to sub-Saharan Africa and some Caribbean islands [27, 28]. Heartwater is severe in exotic and malnourished or stressed local breeds of cattle, and high losses are also observed in naïve local small ruminants and cattle that have been moved to an area in which the disease is endemic [28].

Eleven species of ixodid tick parasitize cattle in Maputo Province, namely *Amblyomma hebraeum*, *Hyalomma rufipes*, *Ixodes cavipalpus*, *Rhipicephalus appendiculatus*, *Rhipicephalus evertsi evertsi*, *Rhipicephalus (Boophilus) microplus*, *Rhipicephalus simus*, *Rhipicephalus kochi*, *Rhipicephalus longus*, *Rhipicephalus pravus* group, and *Rhipicephalus turanicus* [29]. Of these, *A*. *hebraeum* is the main transmission vector for *E. ruminantium*, while *R*.* (B.) microplus* is the main vector for *A*. *marginale*, *B*. *bigemina*, and *B. bovis*. Lastly, *R*. *appendiculatus* is the main transmission vector for *T*. *parva*, although other tick species can still transmit *Anaplasma* species, particularly *A. marginale* [21, 30].

Despite their economic importance, information about ticks and TBDs in the country remains fragmented and incomplete, making reasonable disease control methods difficult to implement. Concerning cattle, official records show that only one study detailing the genetic diversity of *Anaplasma* spp. [1] and another one on *E. ruminantium* [3] were carried out in the country. Therefore, the present study aims to contribute to a better knowledge of the molecular epidemiology of *Anaplasma* species and *E. ruminantium* that infect cattle in four districts of the northern region of Maputo Province.

## Material and methods

### Sampling

**Fig. 1:**
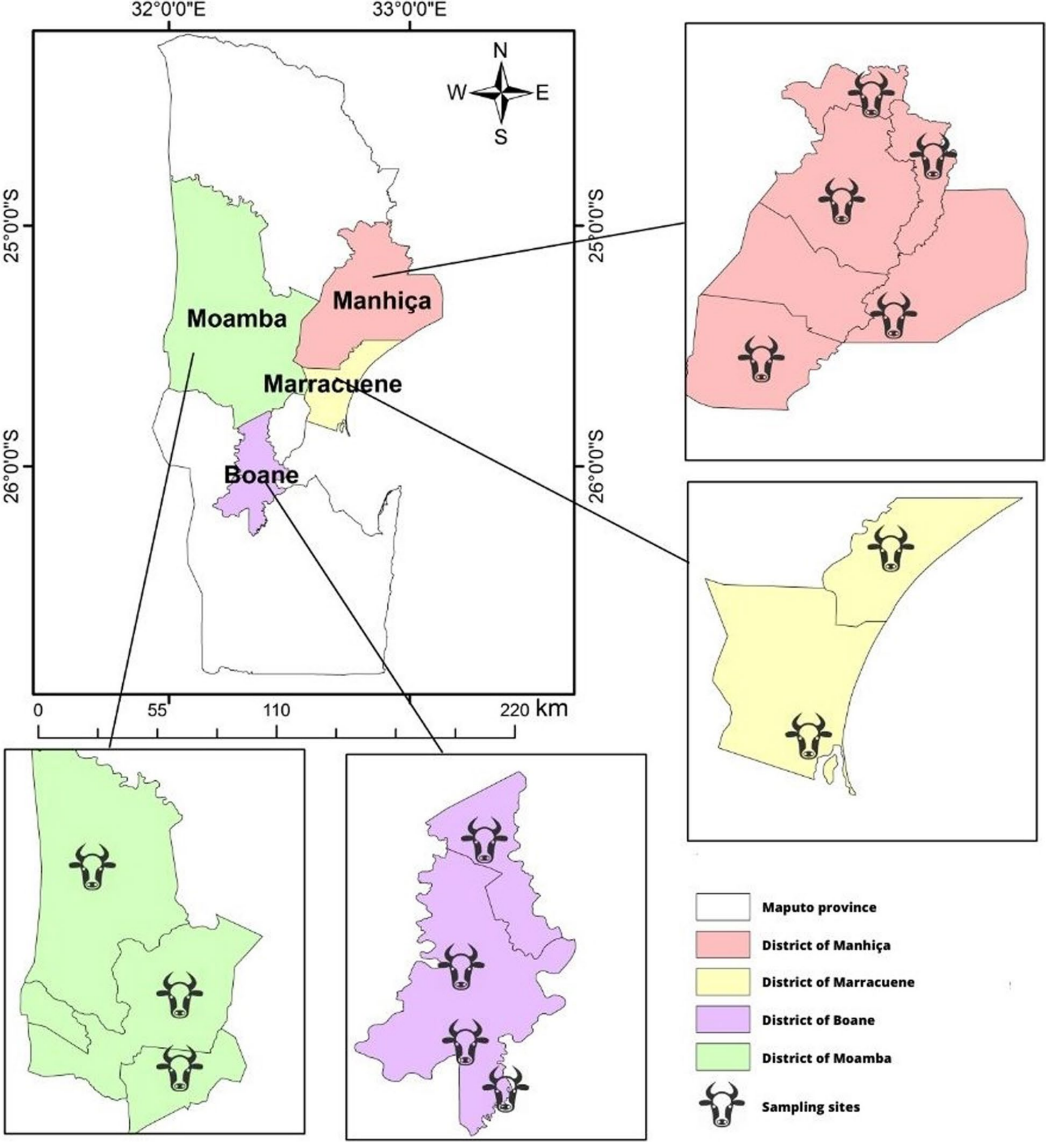
Sites of cattle blood sample collection in Maputo Province, Mozambique between April and September of 2022. Source: Prepared by authors

Between April and September 2022, 200 EDTA-blood samples were collected, by convenience, from apparently healthy adult cattle in four districts of Maputo province, Mozambique **(**Fig. 1**)**. Fifty samples were collected in each of the four selected districts: Boane, Moamba, Marracuene and Manhiça. All the cattle sampled were Nguni and Nguni crossbreeds. Approximately 2–5 mL of blood was collected from the coccygeal vein into Ethylenediamine Tetra-Sodium Acetic Acid (EDTA)-buffered vacutainer tubes. The samples were kept on ice until they arrived at the laboratory, and then stored at -20 °C until analysis.

### Ethical approval

All the procedures were carried out according to ethical guidelines for the use of animal samples permitted by the Institutional Animal Care and Use Committee (IACUC) of the Direcção Nacional de Desenvolvimento Pecuário, Maputo, Mozambique (License number: 161/MADER /DNDP/340/2023). The managers of the surveyed farms were informed about the study and gave their verbal approval prior to the cattle sampling.

### Polymerase chain reaction

#### DNA extraction and molecular detection of *Anaplasma spp.* and *Ehrlichia spp.*

DNA was extracted from 200 μL of each blood sample using the DNeasy® Blood & Tissue Kit (Qiagen®, Valencia, CA), according to manufacturer’s instructions and stored at − 20 °C until its use in amplification reactions.

All DNA blood samples were subjected to a nested PCR targeting a 524 bp fragment of the 16S rRNA gene of *Anaplasma* spp. and *Ehrlichia* spp. as previously described by Rar et al. [11], using primers for initial reactions Ehr1 (5′-GAA CGA ACG CTG GCG GCA AGC-3′) and Ehr2 (5′-AGT A (T/C)C G(A/G)A CCA GAT AGC CGC-5′), and primers for nested reactions Ehr3 (5′-TGC ATA GGA ATC TAC CTA GTA G-3′) and Ehr4 (5′-CTA GGA ATT CCG CTA TCC TCT-3′).

All positive samples in the previously mentioned PCR assay were subjected to a semi-nested PCR targeting a 458 bp fragment of *A. marginale* major surface protein 5 (*msp5*) gene as previously described by Singh et at. [25], using Amar *msp5* eF: GCATAGCCTCCGCGTCTTTC and Amar *msp5* eR: TCCTCGCCTTGGCCCTCAGA as external primers and Amar *msp5* iF: TACACGTGCCCTACCGAGTTA and Amar msp5 eR as internal primers.

In addition, all samples were subjected to semi-nested PCR assays targeting a fragment (720–738 bp) of the *E. ruminantium* Major Antigenic Protein 1 (*map* 1) gene as previously described by Matos et al. [3] using the following primers: External forward primer (ERF3) 5´- CCAGCAGGTAGTGTTTACATTAGCGCA-3´; External reverse (ERR1) 5´-CAAACCTTCCTCCAATTTCTATACC-3´; internal reverse (ERR3) 5´-GGCAAACATCAAGTGTTGCTGATGC-3´. Thus, the external forward primer (ERF3) in the first round of PCR was also maintained in the second round of amplification*.* Amplification reactions were performed in a conventional thermocycler (Gene Amp® PCR System Applied biosystems, Singapore).

Blood DNA samples positive to *Anaplasma* sp. (MH165337), *Anaplasma marginale* (MH124566), and *E. ruminantium* (KY860579), respectively, obtained from naturally infected cattle [1, 3], and ultra-pure sterile water were used as positive and negative controls, respectively.

PCR products were electrophoresed on 1.5% agarose gels to check the size of amplified fragments by comparison to a DNA molecular size marker (100 bp DNA ladder; Promega).

### Sequence and phylogenetic analyses

The amplicons obtained from *Anaplasma* spp. 16S rRNA, *A. marginale msp5*, and *E. ruminantium map*1-based PCR assays showing high intensity of the bands (strongly positive) of expected sizes were purified with Wizard® SV Gel and PCR Clean-Up System Ref A 9282 (Promega, United States) according to the manufacturer's recommendations. Purified amplified DNA fragments were submitted to LGC Genomics, Berlin, Germany for bidirectional DNA sequencing. Consensus sequences were obtained through the analysis of electropherograms using the Phred-Phrap program [31]. The Phred quality score (peaks around each base call) was established at ≥ 20 (99% accuracy of the base call). The Basic Local Alignment Search Tool [BLAST] (http://blast.ncbi.nlm.nih.gov/Blast.cgi) was used to search for homologous reference sequences using the BLASTn algorithm. Alignments of *Anaplasma* spp. 16S rRNA, *A. marginale msp5* and *E. ruminantium map* 1 sequences were constructed, and manually edited using BioEdit (version 7.0. 2.5) program [32].

The phylogenetic analysis was performed using the Maximum Likelihood (ML) method, inferred with RAxML-HPC BlackBox (7.6.3.) [33] and performed in CIPRES Science Gateway [34]. The Akaike Information Criterion (AIC) available on MEGA v. 5 software [35] was applied to identify the most appropriate model of nucleotide substitution. The JC model was chosen as the most appropriate for the phylogenetic analysis of the 16S rRNA, TN93 for the phylogenetic analysis of the *msp5*, and GTR + G evolutionary model for the phylogenetic analysis of the *map1* nucleotide sequence alignment.

## Results

### Detection of *Anaplasma spp.* and *Ehrlichia spp.* in cattle blood DNA samples

Among the 200 cattle blood DNA samples analyzed in the present study, 153 (76.5%) were positive in the PCR targeting the 16S rRNA of the *Anaplasma* genus. On the other hand, none of the samples were positive for *Ehrlichia* 16S rRNA. In addition, out of the 153 cattle DNA samples, 142 (71%) were positive for *A. marginale* as well as the 200 cattle DNA samples, 19 (9.5%) were positive for *E*. *ruminantium*, respectively. Co-infections with *A. marginale* and *E*. *ruminantium* were recorded in 19 (9.5%) samples **(**Table 1**)**. Both tick-borne agents, *A. marginale* and *E*. *ruminantium*, were detected in all four districts investigated. The rate of infection of *A. marginale* and *E*. *ruminantium* varied among sampling locations, ranging from 66 to 76% for *A. marginale*, with an overall occurrence of 71%; for *E*. *ruminantium*, it ranged from 2 to 18%, with an overall occurrence of 9.5%.

**Table 1:** Positivity rate of *Anaplasma* spp., *A. marginale* and *E. ruminantium* in blood samples from cattle in four districts of Maputo province, Mozambique based on nested PCR and semi-nested PCR

	District				
	Boane	Moamba	Manhiça	Marracuene	Total
**Breed**	Nguni	Nguni	Nguni	Nguni	
**N° of sample**	50	50	50	50	**200**
***Anaplasma* sp. (16S rRNA)**	37 (74%)	36 (72%)	38 (76%)	42 (84%)	**153 (76.5%)**
***A. marginale* (msp5)**	35 (70%)	33 (66%)	36 (72%)	38 (76%)	**142 (71%)**
***E. ruminantium* (map1)**	4 (8%)	9 (18%)	5 (10%)	1 (2%)	**19 (9.5%)**
***A. marginale* and *E. ruminantium***	4 (8%)	9 (18%)	5 (10%)	1 (2%)	**19 (9.5%)**

A very low proportion of positivity for *E*. *ruminantium* was reported in Marracuene district (2%) (Table 1). Among the amplified fragments 17 representative sequences of *Anaplasma* sp. 16S rRNA, 19 *A. marginale msp5* and 10 *E*. *ruminantium map1* genes derived from this study were submitted to GenBank database and assigned accession numbers OP297676—OP297692, OQ282861—OQ282879 and OP271794—OP271803 respectively (Table 2).

**Table 2:** GenBank accession number for *Anaplasma* sp. 16S rRNA, *Anaplasma marginale msp*5 and *E*. *ruminantium map*1 genes sequences identified in this study

Animal	Animal ID	GenBank accession number		
		16S rRNA	*msp5*	*map1*
**Cattle**	B5	OP297676	OQ282861	OP271794
	B11	OP297677		
	B37	OP297678	OQ282862	
	B38			OP271795
	B44	OP297679	OQ282863	
	B48	OP297680	OQ282864	
	Ma3	OP297681	OQ282865	
	Ma6			OP271796
	Ma19	OP297682	OQ282866	OP271797
	Ma22			OP271798
	Ma24	OP297683	OQ282867	
	Ma37	OP297684	OQ282868	
	Ma50	OP297685	OQ282869	
	Mo13	OP297686	OQ282870	
	Mo16			OP271799
	Mo19			OP271800
	Mo20	OP297687	OQ282871	OP271801
	Mo27	OP297688	OQ282872	OP271802
	Mo41	OP297689	OQ282873	
	Mo48		OQ282874	
	Mr3		OQ282875	
	Mr5	OP297690	OQ282876	
	Mr11		OQ282877	
	Mr16	OP297691	OQ282878	
	Mr17	OP297692		
	Mr27		OQ282879	
	Mr39			OP271803
**Total**		**17**	**19**	**10**

### Sequences analysis

#### *Anaplasma spp*-16S rRNA sequences

According to BLASTn analysis, the nucleotide sequences of *Anaplasma* spp. obtained in this study are divided into two main groups.

The first group was composed of six nucleotide sequences, two sequences (OP297676 and OP297679) from Boane district, two sequences (OP297683 and OP297685) from Manhiça district, one from Moamba district (OP297689), and one from Marracuene district (OP297691) respectively. These sequences shared 99.5% identity with published sequences of *A. platys* from Saint Kitts and Nevis (CP046391) and from Vietnam (MH686049). The same six sequences also shared 99.5% identity with two published nucleotide sequences of ‘*Candidatus* Anaplasma camelli’ from Iran (MK726038) and Saudi Arabia (KF843827), as well as 99.4% identity with ‘*Candidatus* Anaplasma cinensis’ from China (MH762079) and South Africa (MK814448).

The second group was composed of 11 nucleotide sequences (OP297677, OP297678, OP297680, OP297681, OP297682, OP297684, OP297686, OP297687, OP297688, OP297690 and OP297692), which shared identities ranging from 99.5 to 99.8% with different published sequences: *A. marginale* (MK804764) from Cuba, *A. ovis* (AF309865) from the USA, *A. centrale* (MF289480 and MH588232), from China and Iraq. Finally, six nucleotide sequences of this group, three from Boane district (OP297677, OP297678, and OP297680), one from Manhiça district (OP297682) and two from Moamba district (OP297687 and OP297688) shared 99.5% identity with *A. phagocytophilum* from India (DQ648489). The identity among these 17-nucleotide sequences of *Anaplasma* spp. ranged from 98 to 100%, with query coverage ranging from 99 to 100%.

#### *Anaplasma marginale *- *msp*5 sequences

Nineteen nucleotide sequences of *msp*5 obtained in this study shared identity ranging from 99.78 to 99.55% with sequences of *A. marginale* detected in Sri Lanka (LC467711) and Thailand (MK188829). These sequences showed query coverage ranging from 94 to 100%.

#### *E. ruminantium* - *map*1 sequences

The BLASTn analysis of *E. ruminantium* nucleotide sequences is summarized as follows: Two sequences from this study (OP271794 and OP271797) shared 96.1% identity with two published sequences (JX486794 and JX477668) from Cameroon. The other two sequences of this study (OP271799 and OP271802) shared 100% homology with two published sequences detected in animals from Mozambique (KY856827 and KY860588). One nucleotide sequence from Boane district (OP271795) showed 100% nucleotide sequence identity with one published sequence (AB818942) from Uganda.

Two-nucleotide sequences from Manhiça district (OP271796 and OP271798) shared identities ranging from 99.7% to 100% with sequences (CP063045 and CP040120) detected in South Africa. Three nucleotide sequences (OP271800, OP271801 and OP271803) obtained in this study shared identities ranging from 89.3 to 99.85% with published sequences (CP063043) from South Africa and two sequences (AB818944 and AB818943), both from Uganda. The identity among *E. ruminantium*-map1 nucleotide sequences obtained in the present study ranged from 85 to 100%, with query coverage of 95 to 100%.

### Phylogenetic analysis

**Fig. 2:**
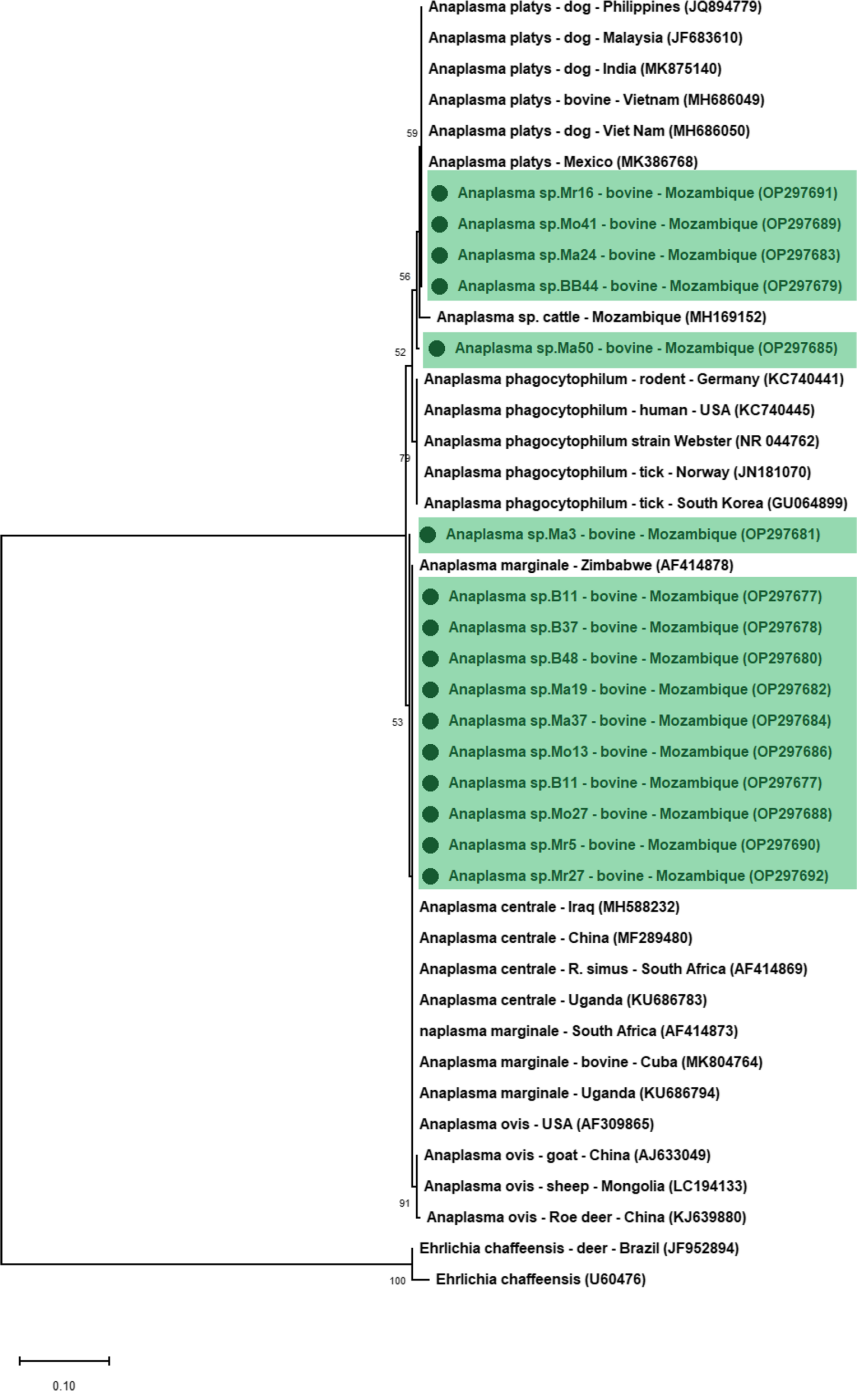
Phylogenetic relationships within the *Anaplasma* genus based on the 16S rRNA region. The tree was inferred by using the Maximum Likelihood (ML) with the JC model. The sequences detected in the present study are highlighted. The numbers at the nodes correspond to bootstrap values higher than 50% accessed with 1,000 replicates. *Ehrlichia caffeensis* was used as an outgroup

In the phylogenetic tree based on the 16S rRNA gene of *Anaplasma* spp., four nucleotide sequences (OP297679, OP297683, OP297689 and OP297691) detected in this study were positioned near to *A. platys* sequences. In addition, one amplified sequence (OP297685) was more closely related to *Anaplasma* sp. previously detected in cattle from Mozambique. The remaining 11 nucleotide sequences (OP297677, OP297678, OP297680, OP297681, OP297682, OP297684, OP297686, OP297687, OP297688, OP297690 and OP297692) grouped together with *A. marginale* and *A. centrale.* All clusters were supported by bootstrap values of 50% (Fig. 2).

**Fig. 3:**
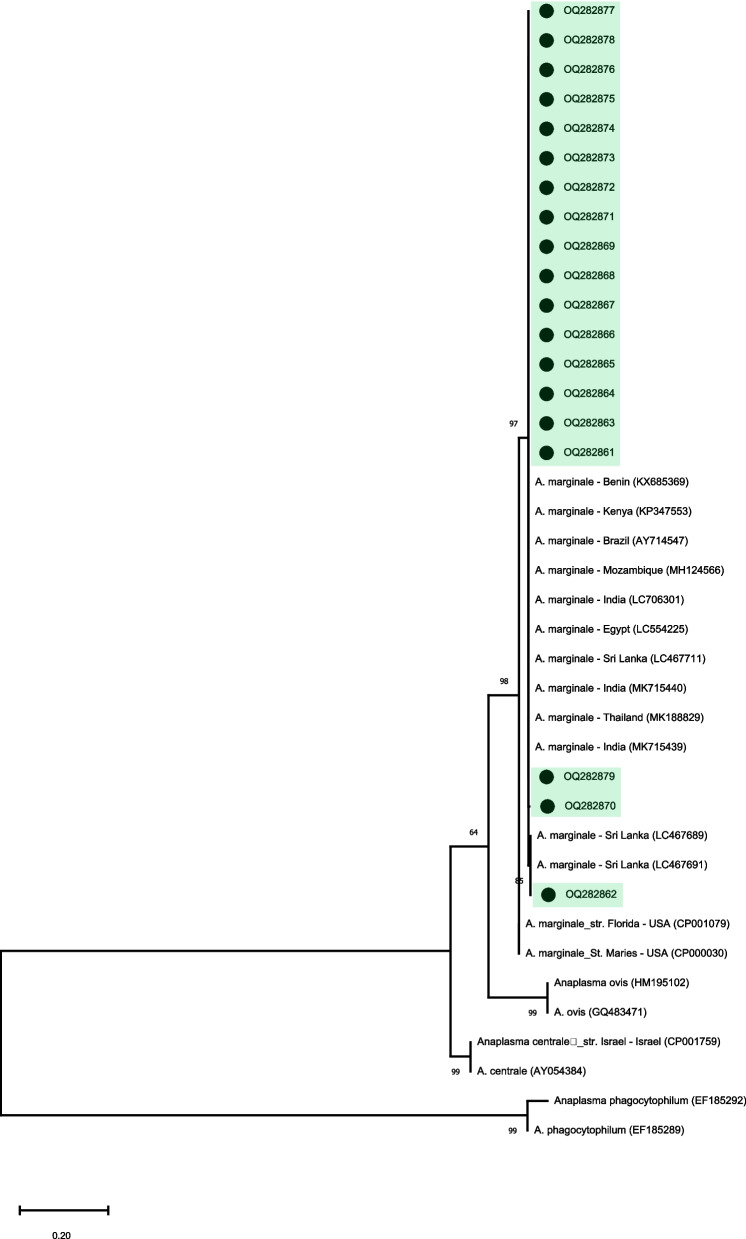
Phylogenetic relationships among *A. marginale* *msp-5* sequences. The tree was inferred by using the Maximum Likelihood (ML) with the TN93 model. The sequences detected in the present study are highlighted. The numbers at the nodes correspond to bootstrap values higher than 60% accessed with 1,000 replicates. *Anaplasma phagocytophilum* was used as an outgroup

The phylogenetic tree based on the *A. marginale*-*msp*5 gene positioned all the amplified sequences in the same main group and clustered with other *A. marginale* sequences from different countries (Fig. 3).

**Fig. 4:**
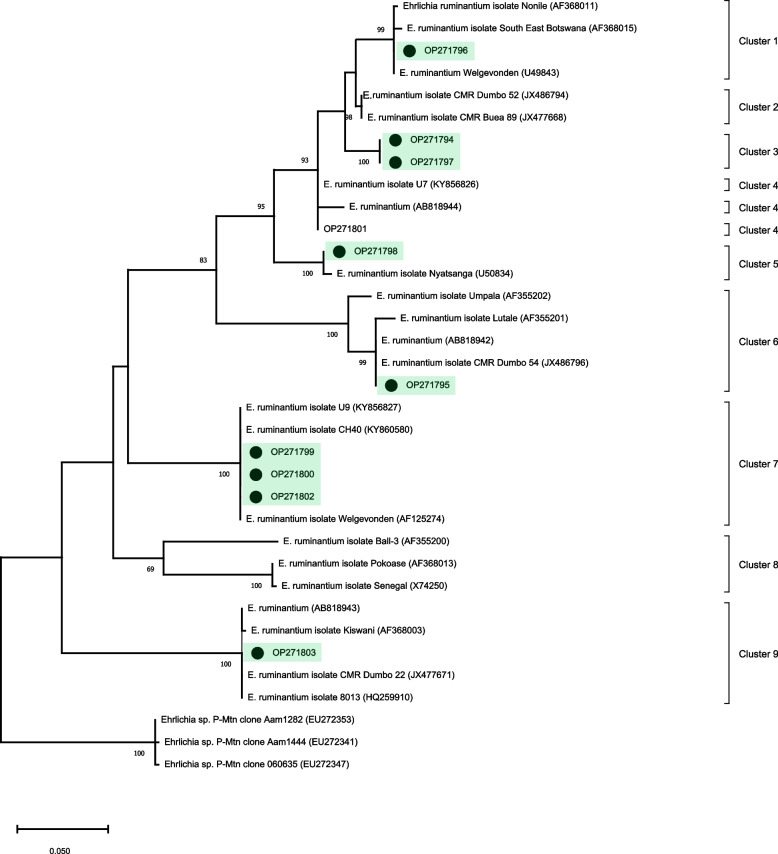
Phylogenetic relationships among the *Ehrlichia ruminantium map1* sequences. The tree was inferred by using the Maximum Likelihood (ML) with the GTR + G model. The sequences detected in the present study are highlighted. The numbers at the nodes correspond to bootstrap values higher than 60% accessed with 1,000 replicates. *Ehrlichia* sp. was used as an outgroup

Finally, the phylogenetic tree based on *E. ruminantium*-*map*1 nucleotide sequences obtained in this study and those retrieved from GenBank clustered into nine clusters. The sequences obtained in this study were positioned in seven different clusters (#1, #3, #4, #5, #6, #7 and #9). In cluster #1, one Mozambican sequence (OP271796) is grouped with three sequences: one from Southern Africa (AF368011), one from Botswana (AF368015) and the last one from South Africa (AF368011). Cluster #3 was formed exclusively by two Mozambican nucleotide sequences (OP271794 and OP271797) obtained in the present study. Cluster #4, two Mozambican sequences, one obtained in this study (OP271801) is grouped with one sequence from Uganda (AB818944). Cluster #5 was formed by two nucleotide sequences: one Mozambican sequence (OP271798) obtained in this study and another one from South Africa (U50834). In the cluster #6, one Mozambican sequence (OP271795) is grouped with four sequences: one from Southern Africa (AF355202), one from Zambia (AF355201), one from Uganda (AB818942), and finally one sequence from Cameron (JX486796). Cluster #7 was formed by six nucleotide sequence. Among them, five from Mozambique, including three obtained in this study (OP271799, OP271800 and OP271802) and another one from South Africa (AF125274). In cluster #9, one Mozambican sequence (OP271803) is grouped with four sequences: one from Uganda (AB818943), one from Tanzania (AF368003), one from Cameroun (JX477671) and one nucleotide sequence from Namibia (HQ259910). All clusters were supported by bootstrap values of 69–100% **(**Fig. 4**)**.

## Discussion

In the present study, the occurrence and phylogenetic relationships of important tick-borne pathogens in four districts of Maputo province were determined and analyzed.

In this study, an infection rate of 76.5% (153/200) of *Anaplasma* spp. was obtained using a nested PCR protocol based on the 16S rRNA gene. Similarly, Fernandes et al. [1] reported an occurrence of 87.2% (191/219) among cattle in the south region of Maputo Province, while Machado et al. [36] recorded an infection rate of 67% (65/97) of *Anaplasma* spp. in African buffaloes (*Syncerus caffer*) from Sofala province, in the central region of Mozambique. In this study, *Anaplasma* spp. phylogenetically associated with* A*. *marginale*, *A*. *centrale*, *and A. platys* were detected. In a previous study carried out in the southern region of Maputo Province, the DNA sequences obtained in cattle were phylogenetically related to *A. marginale*, *A. centrale*, *A. phagocytophilum*, *A. platys*, *A. ovis*, and *‘Candidatus* Anaplasma boleense’ [1].

Based on 16S rRNA sequences, different species of *Anaplasma* might be simultaneously infecting cattle sampled in the current study. Despite the 16S region being conserved, the utilization of this target was important to show the diversity of *Anaplasma* species occurring in cattle from Mozambique. These findings reinforce the relevance of using species-specific PCRs for the detection of *Anaplasma* species to better assist in the conclusion of a diagnosis and the conduction of epidemiological surveys [36].

In Mozambique, particularly in the southern region of the country, cattle farmers also have a pack of dogs that accompany these ruminants to grazing areas, and those animals co-habit with each other, resulting in those cattle being infected by *A. platys*, the causative agent of infectious cyclic thrombocytopenia in dogs, so infection of cattle with this agent should not come as a surprise.

In this study, *Anaplasma* spp. phylogenetically associated with *A. platys* were detected. *Anaplasma platys* has been considered an emerging *Anaplasma* species whose clinical disease is yet to be described [37, 38]. Previous studies in Algeria [15], Mozambique [1], Senegal [38] and Tunisia [39] similarly itemized this pathogen in cattle. Yang et al. [40] suggested the possibility of domestic ruminants acting as alternative hosts or reservoirs for *A. platys*, which is typically a canine pathogen [41]. Therefore, the detection of this pathogen in cattle raises questions of host specificity, as earlier speculated [42]. Zobba et al. [37] noted that several domestic ruminants can harbor a number of strains of *A. platys*, although these strains have different cell tropisms compared to those infecting dogs. The ruminant strains infect neutrophils and are thought to be the ancestral pathogens that evolved to adopt to the canine platelets instead [37, 42]. Previous studies have recognized the zoonotic potential of *A. platys,* which can cause human disease characterized by headaches, intermittent edema, and muscle pains [43]. Consequently, more epidemiological studies are needed to determine the occurrence and clarify the zoonotic potential of *A. platys* in Maputo Province.

The MSP5 is a highly conserved 19-kDa protein and encoded by a single-copy 633 bp gene among *A. marginale* isolates, making it ideal for use in the molecular diagnosis of infection by this agent [25, 44, 45]. The *A*. *marginale* positivity rate of 71% (142 /153) based on the *msp*5 gene fragment detected in this study was lower than the recently reported 97.3% (213/219) based on a qPCR assay targeting the *msp1β* gene of *A*. *marginale* in Maputo Province [1]. This difference might be explained by the fact that the positivity rates of *Anaplasma* spp. might vary according to the diagnostic methods used [46]. On the other hand, reports indicate that the genus *Anaplasma* with causal agents of anaplasmosis in cattle had a higher prevalence in the SADC countries, and *A. marginale* was the most prevalent species of *Anaplasma* [7].

The high positivity rate of *A. marginale* observed in this present study and that reported in a study previously [1] warrants further investigation to evaluate the impact and diversity of this Anaplasmataceae agent on livestock production.

In the present study, *E. ruminantium* DNA was detected in all four searched districts with an overall proportion of infection of 9.5% (19/200), based on a *map*1-nested PCR assay. In a previous study, a positivity rate of 15% (31/210) was obtained according to the pCS20-nested PCR assay [3]. The positivity rate obtained here is relatively lower than that obtained in the previously performed study, and this variation in the positivity rate is probably due to the diagnostic methods used. The pCS20 gene is specific for *E. ruminantium* and is the most sensitive of the probes used for *E. ruminantium* detection, but it is not able to distinguish among the different genotypes. The *map*1 gene has also been used for the diagnosis and characterization of different genotypes of parasites [47]. The infection rate recorded in the present study is, however, sufficiently high to warrant the implementation of appropriate control strategies since clinical disease would be a risk if susceptible animals are present [21, 48]. We can assume that the *E. ruminantium*-*map*1 nucleotide sequences gained from the blood of cattle in the Maputo province were not conserved based on the results of the BLASTn and phylogenetic analyses.

The genetic diversity of *E. ruminantium* constitutes the main limitation for African countries to develop an efficient vaccine [3, 49–51]. Six DNA sequences, of which three from Moamba district were obtained in this study and three other nucleotide sequences obtained in a previous study [3] from three different localities in Maputo Province, shared identity or clustered with the Welgevonden sequence, one of the strains tested for vaccine development. Considering that five new genotypes were identified in this present study, these findings may help to improve current vaccine development and are also vital in understanding the epidemiology and control of heartwater disease.

Further research involving a large population of cattle, goats, and vectors in Mozambique is recommended in order to accurately determine the prevalence, geographic distribution, and genetic diversity of *Anaplasma* spp. and *E. ruminantium* throughout the country.

## Conclusions

The present work indicates that cattle in Maputo Province are a reservoir for multiple Anaplasmataceae species. The 16S rRNA sequences of *Anaplasma* obtained were phylogenetically related to *A. platys* and *A. marginale/A. centrale*. The high positivity rate of infection by *A. marginale* in cattle observed in this present study warrants further investigation to evaluate the impact and diversity of this agent. High genetic diversity of *E. ruminantium* was observed, and five new genotypes of *E. ruminantium*-*map*1 were identified in cattle from Maputo province.

## Data Availability

The *Anaplasma* sp. 16S rRNA, *Anaplasma marginale msp*5 and *E*. *ruminantium map*1 sequences derived from this study were submitted to GenBank database and assigned accession numbers: OP297676-OP297692, OQ282861-OQ282879 and OP271794-OP271803, respectively.
